# Comparative Dynamics of Individual Ageing among the Investigative Type of Professionals Living in Russia and Russian Migrants to the EU Countries

**DOI:** 10.3390/ejihpe10030055

**Published:** 2020-07-26

**Authors:** Tatiana N. Berezina, Natalia N. Rybtsova, Stanislav A. Rybtsov

**Affiliations:** 1 Department of Scientific Basis of Extreme Psychology, Moscow State University of Psychology and Education, 127051 Moscow, Russia; 2 Center for Regenerative Medicine, University of Edinburgh, Edinburgh EH8 9YL, UK; rybnat@yahoo.com (N.N.R.); srybtsov@ed.ac.uk (S.A.R.)

**Keywords:** individual ageing, investigative type of professions, biological age, subjective age, migration, occupational health

## Abstract

The goal of this study was to uncover the influence of professional activity, migration, and gender on dynamics of subjective age and ageing biomarkers. We examined the representatives of investigative types of professions (ITP), 30–75 years old in Russia, (101/62 women), and Russian migrants to the European Union, (101/56 women). ITPs appeared to be ageing slower than statistical standards; men age faster than women; the pre-retirement group (51–65 years old) showed acceleration of relative biological ageing in the Russian sample (women +4.5 years, men +10.7 years) against the EU sample, suggesting a boost of pre-retirement stress in Russia; subjectively, Russian people (51–65 years old) feel close to their chronological age, while EU people perceive themselves far below their calendar age (men—lower by 20.4, women—lower by 10.9 years). The subjective ageing depends on the country of residence, while biological ageing depends on occupation, gender, and negative expectations of retirement.

## 1. Introduction

Ongoing retirement reforms in many countries result in an extended employment period and delayed retirement of a significant number of elderly people; which stimulates the development of healthy active ageing policies [1,2]. An increase of the retirement age is accounted with successes of public medicine in the rise of average life expectancy; the slowdown of individual ageing in Russia; in the European Union; and worldwide [3,4,5,6]. Individual ageing estimation comprises an individual’s psychological and biological age evaluation [7]

Various environmental, genetic, and personality factors affect the rate of ageing, so that people of the same calendar age would be having different biological and psychological age [8,9,10,11,12]. There are several methods that can be used for measuring biological and psychological age to evaluate the relative individual ageing [13,14]. Biological age can help estimate the rate of age-related changes in various body systems such as telomerase activity and telomere length [15,16], age-related measurements of the brain (the "brain age") [17], appearance of clinically significant age-related neurological syndromes [18]. Biological age correlates with physical condition of an individual (especially the state of their cardiovascular system), their life expectancy, and the likelihood of premature death [19]. These indicators are often used by researchers in some countries for empirical studies of relative ageing of various professional groups [20].

In recent years; researchers have increasingly used the indicator “subjective age”—reflecting individual’s health; well-being; their attitude to their profession; as well as themselves [21,22]. Subjective individual age evaluation methods are also used for empirical studies of relative ageing [13,23]. An increase of subjective age is associated with the risk of hospitalization: people with higher subjective age are more likely to get ill [23]

Multidimensional scales are often used for evaluation of subjective age. Such approach considers a person’s assessment of various areas of their life (feelings, interests, look, cognitive activity, personal and working relationships and interactions, and various personality features) [21]. In Russia, a model for assessing subjective age by four factors has become widespread: biological, emotional, social, and intellectual components [24]. Using this approach, there have been many recent studies of subjective ageing of older people [25,26].

The pace of biological and psychological ageing is unique to each person and depends on many factors discussed above. Some of these factors are individual and depend more on a person: this includes healthy behavior, education, the choice of profession, and occupational activity throughout their life [27,28,29]. Other factors are environmental and less dependent on personal efforts [30,31].

In this study, we consider three important factors that influence the rate of individual ageing: (1) the nature of the work and profession, (2) the country of residence and the features of its social policy, (3) the personal efforts that an individual applies to overcome life challenges.

The first factor is the nature of the work and profession. Studies in many countries have shown that the representatives of investigative types of professions (ITP) have a longer lifespan, higher average life expectancy, and a lower probability of premature death [32]. According to J. Holland, investigative career types include professions related to research primarily in scientific fields: academics in any exact or natural sciences, university teachers, employees of scientific laboratories, and design-engineering departments, inventors, editors of scientific journals, etc. Representatives of investigative career types usually have higher education and a scientific degree, conduct research, and have publications in scientific journals [33]. In other research, professionals of this group are often included in larger samples: office workers (as against laborers) [34], people with higher education (as against less-educated workers) [35], white-collar workers (as against blue-collar workers), etc.

Data from studies of public service employees has shown that higher social status, managerial work, and career growth contribute to an increase in life expectancy. The high-level administrators have the lowest probability of premature death, while it is 1.6 times higher for other managers and qualified specialists, 2.2 times higher for office workers, and 2.7 times higher for laborers [34]. Metanalysis of recent articles, mainly from the EU, reported declined risk of early death of employed male ITPs. Men working at jobs requiring cognitive skills have 18% lower early death risk than those who were predominantly involved in manual labor [36]. Furthermore, high level of intelligence and education are directly related to life expectancy and longevity. For example, a study of the average age of death (AAD) of 49.064 famous representatives of investigative and creative types of professions from I century BC until the end of XX century, showed that their AAD is above average. The top five places in life expectancy among men are occupied by Nobel laureates (78.8 years), academics (72.7 years), corresponding members (71.7 years), conductors (71.1 years), and scientists (71.0 years). Among women, the top five places in AAD are occupied by conductors (83.2 years), harpists (80.9 years), academics (80.3 years), harpsichordists (79.1 years), and violinists (78.2 years) [37].

In Russia, several studies of the connection between the investigative type of profession and life expectancy have been carried out. In 1999, Dr. Avgustova studied life expectancy of famous scientists—146 psychologists; she suggests that, on average, representatives of this profession live for quite a long time, and their average life expectancy was 70.5 years while average life expectancy in Russia was 65 years at that time [38]. The study of the demographic characteristics of Russian scientific elite (corresponding and full members of the Academy of Sciences) showed that average life expectancy of full members (mostly men) is 75 years, and 72.1 years for corresponding members (also men) [39], which is higher than the average life expectancy of men in Russia (in 2007, the year of publication, this was 61 years for men). Additionally, the study showed how scientific specialty and scientific work combined with teaching influence the life expectancy of male members of the Russian Academy of Sciences. The minimum mean age of death (MAD) has been found for mathematicians (72.1 ± 0.21 years) and the maximum MAD for scientists in economics (74.6 ± 0.26 years). Scientists who combine research work with teaching at a university or college had a life expectancy of 3.5 years longer than those who were not involved in teaching. The authors concluded that intensive scientific work contributes to an increase in life expectancy and longevity [37].

The second factor is the country of residence. Life expectancy varies in different countries and current life expectancy in Russia is at a global average, while in the EU countries, life expectancy is one of the highest in the world [4,30,40]. Among the modern factors related to the country of residence and affecting the individual ageing of older people is local social and retirement policy. Retirement stress is one of the significant factors impairing health in older age groups [9]. Recent studies in many countries have revealed a post-retirement deterioration of health and increased risk of premature death; this has been shown for Greece [41], Germany [42], Sweden [43], USA [44], and Russia [45].

Retirement stress affects the relative ageing of both ITP and labour type workers. A study of Finnish municipal employees revealed an increase in musculoskeletal diseases among men after retirement: (39–58% against an increase of 25–37% for those who continued working. The authors indicate that the probability of developing diseases is greater for ITP workers than laborers, and greater for men than women [46].

Retirement stress is also described in Russia. However, it begins at an earlier age than in many EU countries due to earlier retirement [6]. Besides, previously we showed that in addition to retirement stress, there is also stress associated with the expectation of retirement, which also leads to health deterioration: in Russia, pre-retirement health deterioration in men begins at the age of 46–50 years, with the highest rate of relative ageing at 56–60 years, after which health indicators gradually return to the age norm. For women, ageing and deterioration of health indicators begins at the age of 51–55 years, reaching its maximum at the age of 56–60 years and persists during the next age stage, between 61–65 years, and then the age norm is restored [45]. At the same time, life expectancy in Russia is significantly affected by both social and professional environment [47]. High intellectual professional achievements contribute to an increase of life expectancy among representatives of high-risk occupations [48,49].

Overall, the data collected in Russia shows that the ITPs have a higher average life expectancy, which implies a slowdown in relative ageing rate and better health indicators in pre-retirement and post-retirement age.

The third factor is personal effort to overcome life challenges. The concept of “the personal organization of time” implies that a person acts as the creator of their life journey. The person is an active subject and can vigorously change their life and destiny. Moreover, when meeting with challenges, the more the person invests and succeeds in their life changing, the more they develop and increase their life resources [50]. ITP migrants from Russia have made more personal efforts to organize their life circumstances. They mastered to learn the language and cultural traditions of a new country, to adjust their education, and apply it to get a job. All of this requires greater contribution of forces than building a career in home country.

We hypothesize that immigration to the EU countries for ITPs is a favourable factor that reduces the rate of individual ageing, especially for people of pre-retirement age.

In this study, we assess the dynamics of biological and psychological age indicators among investigative type professionals of several age groups that have the common country of origin, but differ by country of residence. One part is comprised of people living in Russia, and the other is comprised of professionals who settled in the EU countries at a young age and continue working in their degree field.

## 2. Materials and Methods

To study the dynamics of biological ageing, the method of “age sections” was used. To assess the indicators of biological age, we used the following methods.

Biological age (BA) determination according to V.P. Voitenko [51,52]: this technique was developed at the Gerontology Research Institute of the Academy of Medical Sciences of the USSR and is actively used in Russia (see Supplementary Methods). Formulas for measuring biological age include activity indicators of cardiovascular, respiratory, musculoskeletal, and equilibrium systems as well as of metabolism and psychological indicators (subjective assessment of health) [53]. The method was tested and validated in Russia; it has been shown that biological age determined by this method correlates with the state of cardiovascular system and lifespan [52].

Subjective assessment of health (SAH) decline: this indicator is included in biological age formula (see Supplementary Methods). It is a questionnaire listing the signs of somatic diseases and behavioural characteristics associated with age. The higher the score on the questionnaire, the more age-related somatic symptoms of health decline the test subject has. This method allows separate evaluation of the subjective component of biological age. Subjective assessment of health was also reported as reliable predictor of premature death risk [54,55].

Static balancing (SB): this indicator is also included in biological age formula (see Supplementary Methods). Static balancing, an indicator of the state of musculoskeletal system, coordination of movements, and how determined an individual is to achieve the best result, is one of the objective components of biological age [56,57]. It is measured in seconds, and the test subject is to stand on the left foot until the right foot touches the floor. The test involves standing without shoes, with eyes closed, arms resting at the sides. The time is recorded three times using a stopwatch with an interval of 5 min between each trial. The best result is used.

Expected biological age (EBA) for different age groups: EBA is a statistical indicator that characterizes average biological age within a specific age group currently living in Russia. The EBA was calculated according to the formulas of Dr Voitenko [53]: for men EBA = 0.629 × calendar age (CA) + 18.56; for women EBA = 0.581 × CA + 17.24. To develop this indicator, V.P. Voitenko examined large groups of healthy people of various ages and, using regression equations, derived a formula for estimating the EBA indicator of a healthy person.

1. The relative biological ageing index (biological age minus expected biological age, BA-EBA) allows us to evaluate how much an individual is older than their statistical age norm in regards to their health condition. Negative values indicate individual youthfulness of a person, and positive values indicate individual ageing respective of statistical norms. This is the main indicator used to assess the dynamics of relative ageing.
2. Subjective psychological age (PA), developed in the laboratory of personality psychology of the Institute of Psychology of the Russian Academy of Sciences (authors K.A. Abulkhanova and T.N. Berezina), based on the concept of personal organization of time [24,50] and on analysis of the subjective age of aged individuals [58,59]. The test subjects were asked to evaluate their age at the 100-point scale (from 0 to 100). The test subject may choose any number in this interval, corresponding to the self-esteem of their psychological age. Where 0 point is the psychological age of a new-born baby who has neither life experience nor personality, whose psyche is just beginning to develop. One hundred points is the psychological age of a person completing their course of life, who has achieved everything or will never progress from there, whose psyche is undergoing age-related degradation. The person chose any age in the range from 0 to 100, corresponding to their subjective sensation. We conducted a preliminary comparison of our methodology with the well-known methodology for assessing subjective time [24]. Similar to our 100-point scale was used as well. High levels of agreement were obtained on the total scale of Barack’s subjective age and on the assessment of subjective personality age according to our methodology (according to Pearson’s correlation coefficient). Therefore, for further analysis, we used the results obtained using our methodology.
3. Index of relative psychological ageing (psychological age minus calendar age, PA-CA). Negative values indicate that a person is younger than their calendar age and is looking forward to the future. Positive values indicate that a person perceives themselves as more mature, wise, and successful than other people at this age.
4. Statistical analysis. We tested normal distribution of age indicators. For the subjective age, biological age, expected biological age, and relative ageing index, the deviation from the normal distribution was not significant. Descriptive statistics were also calculated: average and standard deviation. To assess the influence of the country of residence on the indicators of relative psychological and relative biological ageing in men and women, Anova Factorial was conducted, as well as analysis of variance with the assessment of Fisher criteria (Fisher LSD). Anova Factorial analysis was applied to evaluate the significance of the influence of the following factors: the country of residence and gender, on relative ageing, in different age groups. We also calculated the interaction of these factors. For statistical analysis, we used the program Statistica -12 (SoftStat).

Test subjects. The final sample consisted of 202 individuals who are representatives of investigative types of professions according to J. Holland: researchers, employees of research institutes, university lecturers, and professionals in related fields with a scientific degree. The profession of subjects was determined by primary employment. We tested two big groups. One group consisted of ITPs from EU countries: 101 individuals aged between 30 and 75 years (of which 56 women and 45 men). All are immigrants from Russia permanently residing in the EU countries for a period of at least 5 years (average time of residence is 15 years, the shortest period of residence is 5 years, the longest is 30 years). All have EU citizenship or residence permit, and most are considering continuing working there until retirement. Most of them are graduates of major Russian universities. By the time of the survey, most migrants had already changed several countries of residence within the European Union (among them: Belgium, France, Germany, Italy, Netherlands, Norway, Poland, Spain, Sweden, Switzerland, and others). However, the survey was conducted in Scotland (UK) where all respondents were residing at the period of our investigation. Another group were ITPs from Russia: 101 individuals aged between 30 and 75 years (of which 62 women and 39 men). Most of them are also graduates of major Russian universities. Both surveys were carried out from April until September 2019. Both groups were divided into 4 age subgroups: up to 35 years old, 36 to 50 years old, 51 to 65 years old, and above 66. The age and gender composition of the sample is presented in Table 1. In general, the ratio between age groups in the sample of EU countries reflects the actual representation of Russian ITP migrants, and the ratio of age groups within the sample from Russia corresponds to the distribution of ages (according to Russian Federal State Statistics Service data).

After collecting the data, we conducted extensive data quality checks to identify respondents who were not making enough effort. To increase the severity of the analysis and increase the reliability of the results of the study, we used the direct, archival, and statistical screening methods [60]. We excluded participants who missed part of the significant questions in the questionnaire. Participants who gave extreme values in assessing subjective age were asked an additional question to assess the degree of their understanding of the methodology; in doubtful cases, their results were also excluded.

## 3. Results

The effect of the place of residence (Russia or EU countries) on the indicators of relative ageing of the ITPs of two genders is presented in Table 2. As shown in the table, there is no interaction of factors for all variables, i.e., the influence of test subjects’ gender does not depend on the country of residence. In other words, relative ageing can depend on gender and country of residence, but these relations are similar, differing quantitatively, but not qualitatively.

The country of residence significantly affects the following characteristics: relative biological ageing, relative psychological ageing and subjective evaluation/self-assessment of health. Gender, in its turn, only significantly affects relative biological ageing and subjective evaluation of health; therefore, relative psychological ageing of ITPs in Russia and the EU does not depend on their gender. The indicator of static balancing is not dependent on either the country of residence or gender of the participants. The data on relative biological ageing of ITPs in Russia and the EU countries is presented in Table 3.

The differences for genders are among the most pronounced (Table 3). Relative biological ageing is less pronounced in women than in men, i.e., women have lower relative biological age indicators than men. For the Russian sample, this can be seen in all age ranges, and for EU countries only in the age range of up to 35 years and between 36–50 years old; men and women in older groups age at a similar rate.

Differences in relative biological ageing based on country of residence appear only in 3 groups: between men aged 51–65 years (significant), and between women aged 35–50 years and 51–65 years (differences in both groups at the tendency level only). In all different age groups in the EU sample, the ageing of researchers is slower.

The table also shows an increase in relative biological ageing in the age interval from 51 to 65 years for both men and women of ITPs. At younger ages, participants living in Russia show tendency for slower relative biological ageing—this indicator is negative and does not exceed statistical standards [53]. At an older age, relative biological ageing also significantly slows down. Such a pattern was not observed in the EU sample. The differences in indicators of static balancing and subjective assessment of health in Russia and in the European Union are presented in Table 4.

There are no significant differences in static balancing (the objective indicator of biological age) for different genders or country of residence. However, according to subjective indicator of biological age—the subjective self-assessment of health decline—the differences are significant, based on both country of residence and genders (Table 4). Women find significantly more age-related ailments and behavioural characteristics than men, which happens both in Russia and in the EU countries. At the same time, the indicators of health decline are lower among male and female participants living in EU countries, i.e., they find fewer age-related problems than similar representatives of the ITPs living in Russia. The dynamics of relative psychological ageing in Russia and the EU countries is presented in Table 5.

Differences between countries and age groups are more pronounced than differences between genders (Table 5). Relative psychological ageing is more obvious in Russia than in EU countries. Russian ITPs tend to consider/perceive themselves older, more successful and wiser than their calendar age would allow. EU residents of Russian origin, on the contrary, tend to consider/perceive themselves younger and less successful and tend to believe that everything is still ahead of them. The differences are significant for women under 35 years old and between 51–65 years old; for women over 65 years old, these are at tendency level. For men, the differences are significant for all groups (except for those over 65—these are at the tendency level).

However, the dynamics of ageing within age groups is similar both in Russia and in the EU. Overall, young people (under 35 years old) tend to overstate their psychological age; their relative psychological ageing rates are the highest and significantly differ from the older groups based in Russia, both for all men and women. With age, the trend gradually reverses, and men and women after 65 are prone to understate their psychological age compared to their calendar age, so their relative psychological ageing is negative, and their hopes for future achievements are still high.

Differences in psychological ageing index between genders are significant only in one group: women from Russia in the age range between 36–50 years are significantly different from men. At this age, men continue to consider themselves psychologically older, wiser, and more successful, while women are already starting to consider themselves younger.

## 4. Discussion

The subjects in the studied samples did not differ much at the beginning of their course of life. These were young people who graduated from universities (mainly prestigious educational institutions in Moscow), obtained professions of the intellectual type. Many graduated with honours and entered a PhD program. Some of them emigrated to continue their education or received a work contract in the EU countries. Other graduates remained home to continue their postgraduate or PhD studies in Russia, found work in research institutes, became university staff or lecturers or engaged with the private sector. They also changed jobs and places of residence, but relocations were limited to the Russian Federation. They had no need to study a foreign culture, language, or to adapt to the laws and customs of another country. In general, they have put fewer personal efforts into building their destiny.

**Figure 1:**
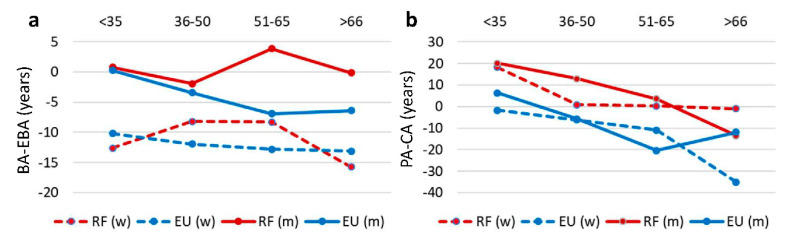
Dynamics of individual ageing indices among ITPs in Russia (RF) and Russian migrants to the EU countries. (**a**) Index of individual biological ageing (BA-EBA). Expected biological age is zero line. (**b**) Index of individual psychological ageing (PA-CA). Calendar Age is zero line. For significance of the differences between the points, see Table 3 and Table 5. For significance of the differences between country of residents and gender and factors interaction, see Table 2. More explanations are in the text. Men (m) are indicated by solid lines, women (w) by dotted lines. Red colour lines—Russian Federation (RF), and blue colour lines—the European Union (EU).

For both genders, men and women under 35 show no significant difference in relative ageing index, between the EU or Russian samples (Table 2, Figure 1a,b). We attribute this to the fact that these respondents have recently immigrated from Russia to the EU (2–10 years ago) and the migration and the subsequent course of life in a new country had no significant impact on their biological age.

However, significant differences in the relative ageing index in the 36–50 years group were found between ITP migrants in the EU and Russian residents. The relative ageing index for both women and men who left for the EU is significantly lower than for their ITP colleagues who remained in Russia. This age group of ITP migrants have been working in the EU for 8–25 years, changed several countries of residence, and most received a residence permit or citizenship. In the age group 51–65 years, people have been living in the EU even longer, everyone has citizenship or a residence permit, and have professional achievements. They differ even more from their Russian counterparts in the relative ageing index. We found the greatest difference in men (51–65 years old) who migrated to the EU is that their biological age is below statistical norms (Table 3, Figure 1a). Men in this age group in Russia, on the contrary, are beginning to accelerate ageing. We attribute this to the ongoing retirement reform in Russia, the main burden of which falls on this age group. Unfortunately, the EU group over 66 is represented by a small number of respondents (Table 1). We managed to engage very few ITP EU-migrants of this age group to attend the survey. Perhaps, some potential respondents over 66 are unavailable because they became more introverted with age, others might have returned to Russia from the EU after retirement due to economic or family reasons [61]. However, we indicated the tendency showing that in this age group the differences in the index of biological ageing between ITP migrants in the EU and in Russia mostly disappear.

We hypothesized that one of the reasons for the differences in relative individual ageing between the EU and Russian samples is the effect of pre-retirement stress, which is more pronounced in Russia due to ongoing retirement reform. The fact of retirement stress has been shown for many countries [9,41,46]. However, earlier we showed that pre-retirement stress (the stress of anticipation of retirement) is more typical for Russia than post-retirement stress (or simply called retirement stress). We also demonstrated that the phenomenon of retirement stress is typical in Russia for almost all professional groups (people with higher education, with secondary education, unemployed, and representatives of high-risk professions) [45]. We described two characteristics of retirement stress. Firstly, the acceleration of biological ageing in the interval between 50–65 years (Table 3, Figure 1a). Secondly, a mismatch of the passage of biological and psychological time within the same age range (Table 5, Figure 1b). Both men and women in Russia at this age tend to consider themselves subjectively younger than their calendar age, while their bodies’ biological age has increased. We explained the phenomenon of retirement stress by the uncertainty which is caused by the retirement reform currently underway in Russia. Retirement reform in Russia sparked a public outcry incomparable with many other countries, which expresses itself/is expressed in public discussions and mostly negative attitude of many Russians to the reform. Retirement stress can also be explained by the unexpectedness of the reform. In psychology, there is a concept of a mindset. If people have known and expected for many years that they will retire at the age of 55–60, and this expectation is suddenly no longer valid, they find themselves in a state of error of nervous processes, of disruption of their mindset. This, in its turn, has a harmful effect on an individual’s adapting to the surroundings as well as on their mental and physical well-being. Especially severe stress levels are found among people of pre-retirement age, as they were the ones expecting the forthcoming retirement, and they are the ones to suffer most from the retirement reform.

A similar uncertainty can be found in any country with an ongoing retirement reform. We compared our data with the indicators of individual ageing of a mixed group of professionals from the Republic of Kazakhstan [20]. Interestingly, the retirement reform for men in Kazakhstan began 20 years ago and is now completed. The current generation of men have already adapted to the increased retirement age. As for women, the reform began a few years ago and nowadays women in Kazakhstan are also in the state of uncertainty. We found that women in Kazakhstan also show signs of retirement stress, which has the same symptoms as observed in women in Russia: in particular the acceleration of biological ageing in the age range of 50–65 years. Such a phenomenon is not observed for men.

The group of ITPs in Russia exhibited only one sign of retirement stress: they demonstrate an acceleration of relative biological ageing in the age group between 5065 years—a period that includes the years preceding the retirement (55.5 years for women and 60.5 years for men) and the years immediately after retirement (Table 3, Figure 1a). A mismatch in the dynamics of their psychological and biological time did not occur (Table 5, Figure 1b). This sign is generally less pronounced for this sample than for professionals of other types living in Russia.

In particular, we previously discovered [20,50] that women in Russia have the following relative ageing indices: at the age of 51–55, this indicator is −0.74, at the age of 56–60 it is 2.0, and at the age of 61–65 years old it is 1.5. For men, the trend is similar. It is worth noting that relative ageing index values among ITPs living in Russia are much lower (−8.3 years). For men from other professional groups, the relative ageing index has the following values: at 51–55 years old it is 4.0, at 56–60 years old it is 4.3, and at 61–65 years old it is 4.0. For ITPs men, the relative ageing index at this age is 3.9.

The second sign of retirement stress was not found among ITPs living in Russia: they do not have a mismatch of psychological and biological age (when the body ages faster biologically, but psychologically people consider themselves younger). On the contrary, the relative psychological ageing index of people under 65 years is positive, i.e., they consider their psychological age to be higher than their calendar age, unlike the representatives of other professional groups in Russia. Perhaps this is because subjective age is the age of psychological and personal maturity, a measure of achievement. It turned out that it is important for ITPs to emphasize their achievements (possibly in comparison to their peers in other types of occupation), thus increasing their subjective age. This differs from the usual tendency of adults in Russia to understate their subjective age against their calendar age (Table 5, Figure 1b).

The second possible reason for the relative biological ageing variation is the country of residence. The ITP respondents who moved to the EU changed several places of residence and do not necessarily bind themselves to any single country with a specific model of retirement scheme. Most of them prefer living in the present rather than thinking about future problems. Perhaps, living in countries more prosperous than Russia has reduced their existential fear of the future.

On the other hand, their biological and psychological age is lower than that of their peers remaining in Russia (Figure 1a,b). The rate of their relative biological and psychological ageing is reduced. In general, this is in line with global trends in life expectancy. Since the average life expectancy in Russia is lower than in the EU countries, it is fitting that Russian residents age faster and EU residents age slower, even if they are migrants from ex-USSR countries. However, we have noticed the dynamics of the subjective and objective components of biological age. Self-assessment of health decline was taken as the subjective component, and static balancing as the objective one. As shown in our study, the difference between the Russians who left for the EU countries and those who remained in their homeland lies in subjective age characteristics. In the other words, the objective indicator of health (time of static balancing) among Russians and migrants does not differ, but according to the subjective assessment of health decline, the differences are at their largest. This can also be confirmed by the dynamics of subjective psychological ageing. This indicator expresses the biggest differences between ITPs living in Russia and in the EU. ITP migrants tend to consider themselves significantly younger in comparison with their biological age. For example, women aged between 51–65 years consider themselves younger by an average of 10 years, and men by an average of 20 years (Table 5, Figure 1b). Therefore, it can be assumed that the difference in the dynamics of relative ageing of investigative occupational types is explained mostly by psychological, not biological factors—these are changes in psychological attitudes, changes in attitude towards oneself, a focus on the social environment different from Russia. In general, this leads to the fact that Russian ITPs who moved to the EU countries begin considering themselves younger, start taking more care of their health and considering it better, even though their objective health indicators may not be as good as those of native Europeans, and they may be less likely to have a longer life expectancy, as previously indicated [4].

## 5. Evaluation of Data and Methods Limitations

This study provided new insights into the rate of the biological and psychological ageing of ITPs. However, some limitations in the data and methods should be considered. Our choice of respondents was guided by voluntary consent to conduct the survey and process the data. Some people refused to take part in the survey, and we are aware that there was an involuntary selection of more proactive respondents (presumably extroverts) and we may have unintentionally excluded certain respondents, such as introverts, from analysis. This might have weakened our results. It is also possible that people who decided to migrate were initially strong and determined, and this possibly explains their greater resistance to stress. Also, their enthusiasm for a change of residence could lead to their ignorance of real problems at new place. Perhaps therefore they experience less stress than their counterparts in home country. However, one cannot exclude that due to changes in the cultural environment and language problems, emigrants do not immediately become full members of society, they are more vulnerable and not adapted to local life. Migrants tend to be in a disadvantaged position in comparison to residents of the country in a similar socioeconomic situation [4,40,62]. At the same time, the remaining in the home country feel wiser since they are adapted well in society and feel less stressed and more protected than migrants into the EU. However, perhaps we were not able to consider all the factors affecting the ageing of ITPs migrated to the EC. Further studies may be needed to reveal whether this result can be indeed widespread to the other groups of population.

## 6. Conclusions

The comparison of the relative individual ageing dynamics of respondents of the intellectual occupational types in Russia and Russian migrants to the EU countries allowed us to identify several general trends and differences. We consider general trends to be: (1) the general dynamics of biopsychological ageing, which largely coincides with the dynamics of calendar age; (2) the differences in relative ageing between men and women: both in Russia and in the European Union, men age faster than women, which applies to both biological and psychological age; (3) in general, among representatives of the ITPs, relative biological ageing is slower than the statistical standard, and is, therefore, slower than for most of their peers of other occupational types; (4) the tendency to exaggerate their psychological age by younger people (under 35 years), and to exaggerate it as they grow older—people over 65 years old tend to psychologically consider themselves younger; this applies to both men and women in Russia and in the EU. We also found differences in the dynamics of relative ageing among representatives of investigative occupational types in Russia and Russian migrants in the EU countries. First: Russian ITPs experience retirement stress that affects their relative biological ageing. In Russia, biological characteristics of health begin to deteriorate for both men and women in the age group between 51–65 years, which leads to accelerated ageing of the body; before and after this age interval, accelerated ageing was not observed. For Russian migrants in the EU countries, retirement stress that affects the individual ageing was not found. Second: biological ageing is more pronounced in Russia than in the EU countries. In almost all age ranges, ITPs who moved to Europe are biologically younger than their peers who remained in Russia. Third: there are differences in the dynamics of psychological ageing of the ITPs. Russian researchers under the age of 65 living in their home country estimate their subjective age roughly equal to the calendar age or slightly higher. Russian migrants, in contrast, tend to rate their subjective age below calendar age: women between 51–65 years old estimate their age lower than calendar age by 10 years, and men by 20 years.

## Figures and Tables

**Table 1:** Gender and age composition of study participants from Russia and the EU countries (number of people).

		Group 1	Group 2	Group 3	Group 4
	Age	Up to 35	36–50	51–65	66+
Russia	Women	13	21	20	8
EU sample	Women	7	30	18	1
Russia	Men	11	14	10	4
EU sample	Men	12	27	5	1

**Table 2:** Influence of the place of residence on the indicators of relative ageing based on gender (Anova factorial results).

Effectors	Place of Residence		Gender		Interaction of Factors	
	F	*p*	F	*p*	F	*p*
Relative biological ageing	5.42	0.021 *	77.78	0.000 *	0.48	0.491
Relative psychological ageing	25.67	0.000 *	3.21	0.075	0.21	0.648
Subjective self-assessment of diseases	11.71	0.001 *	35.78	0.000 *	0	0.969
Static balancing	0.83	0.364	0.92	0.34	0.01	0.908

* *p* < 0.05.

**Table 3:** The comparative analysis of relative biological ageing index (biological age minus expected biological age, BA-EBA) among investigative types of professions (ITPs) in Russia and Russian migrants to the EU countries.

		Group 1	Group 2	Group 3	Group 4
	Age	Up to 35	36–50	51–65	66+
Russia	Women	−12.6	−8.2 *^,4^	−8.3 *^,4^	−15.7 ^2,3^
EU sample	Women	−10.2	−11.9 *	−12.8 *	−13.1
Russia	Men	0.8	−1.9 ^3^	**3.9**^4,^**	−0.1
EU sample	Men	0.3	−3.4	**−6.9** **	−6.4

* The difference between Russia and the EU countries in this age group is at tendency level only (*p* < 0.1). ** The difference between countries in this age group and for this gender is significant (*p* < 0.01). ^1,2,3,4^ age groups under these numbers display significant differences depending on country of residence and gender (*p* < 0.05). The underlined figures indicate results where differences are significant for genders, based on their age range and country of residence.

**Table 4:** Static balancing (SB) and subjective assessment of health (SAH) decline among ITPs in Russia and Russian migrants to the EU countries.

	Residence	SB (in Seconds)	SAH
women	Russia	35.9	9.2 *
	EU sample	31.2	7.1 *
men	Russia	39.8	5.4 *
	EU sample	36.2	3.2 *

* The difference between Russia and the EU countries is significant (*p* ≤ 0.05). The underlined figures indicate results where differences are significant for genders based on their country of residence.

**Table 5:** The relative psychological ageing index (psychological age minus calendar age, PA-CA) among ITPs in Russia and Russian migrants to the EU countries.

		Group 1	Group 2	Group 3	Group 4
	Age	Up to 35	36–50	51–65	66 plus
women	Russia	18.3 **^,2,3,4^	0.9 ^1^	0.3 **^,1^	−1
	EU sample	−1.7 **	−6.2	−10.9 **	−35.0 *
men	Russia	20.1 **^,3,4^	12.9 **^,4^	3.6 **^,1^	−13.5 *^,1,2^
	EU sample	6.3 **^,2,3^	−5.7 **^,1^	−20.4 **^,1^	−12.0 *

* The difference between Russia and the EU countries in this age group is at tendency level only (*p* < 0.1). ** The difference between countries in this age group is significant (*p* < 0.01). ^1,2,3,4^ the age groups under these numbers display significant differences depending on country of residence and gender (*p* < 0.05). The underlined figures indicate results where differences are significant for genders based on their age range and country of residence.

## Supplementary Materials

The following are available online at https://www.mdpi.com/2254-9625/10/3/55/s1.
