# Supplementary material for: Comparative Dynamics of Individual Ageing among the Investigative Type of Professionals Living in Russia and Russian Migrants to the EU Countries
Source: Eur J Investig Health Psychol Educ. 2020 Jul 26;10(3):749–62. doi: 10.3390/ejihpe10030055 (PMC8314298; doi:10.3390/ejihpe10030055)
Supplement: Supplementary file 1 [file ejihpe-10-00055-s001.pdf]

Supplementary methods

Biological and psychological age evaluation (poll and scoring examples)

Summarised, adapted and developed by Abulkhnova K.A., Berezina T.N.

Each respondent was assigned an individual number for blind analysis of the result. The survey was conducted by a trained field scientist while statistical analysis and conclusions were carried out by other researchers. The biological and psychological age evaluation includes measuring of several indicators.

1.**Biological age** determination according to the method of V.P. Voitenko, including 3 indicators: biological age (BA), expected biological age (EBA) and relative aging index (BA-EBA).
2. Self-assessment of subjective **psychological age** according to K. A. Abulkhanova and T.N. Berezina (PA).
3. **Index of relative psychological aging** (psychological age minus calendar age, PA-CA).

1. Biological Age (BA)

First, measure the following indicators and enter them into the formulas below.
1) SBP - Systolic blood pressure (upper).
2) DBP - Diastolic blood pressure (lower).
3) PP - Pulse pressure - the difference between systolic blood pressure and diastolic blood pressure (i.e., upper and lower). This indicator is used for women.
4) BH - duration of Breath Holding after a deep inhale (in seconds). This indicator is needed for men.
5) BW - Body weight (in kg). For women.
6) SB - Static Balancing - determined when the subject is standing on their left leg, without shoes, eyes are closed, hands are lowered along the body, without preliminary training (in seconds). All subjects, both men and women.
7) SAH – Subjective Assessment of Health decline (in points). Answer the test questions and calculate points using the key below. The first 28 questions are answered with "yes" or "no", the last - "good", "satisfactory", "bad" and "very bad".

1. Do you get or have headaches?
2. Can you say that any noise easily wakes you up at night?
3. Do you get chest pains or angina?
4. Do you think your eyesight has deteriorated?

5. Do you think your hearing has deteriorated?
6. Do you drink only boiled or filtered water?
7. Do people give up their sits for you on public transport?
8. Do you have joints pain?
9. Do weather changes affect your well-being?
10. Do you have a hard time sleeping or insomnia due to anxiety?
11. Do you get or have constipation?
12. Do you get pain behind the right, lower rib area (liver area)?
13. Do you have vertigo?
14. Have you found it harder to concentrate recently?
15. Do you have more often memory lapses, forgetfulness?
16. Do you feel burning, tingling or goosebumps?
17. Do you have tinnitis (ringing in the ears)?
18. Do you keep heart drops or similar medicines at home?
19. Do your legs get tired or swollen after you walk?
20. Have you had to give up some food?
21. Do you have shortness of breath when walking fast?
22. Do you get or have pain in your lower back?
23. Do you drink mineral water for medicinal purposes?
25. Can you say that you cry more easily than before?
26. Do you visit the beach?
27. Do you think that now you are as efficient as before?
28. Do you have periods when you feel joyfully excited, happy?
29. How do you rate your health?

Key

Questions 1-25: add 0 points for each “no” answer, for the answer “yes” add 1 point.
Questions 26-28: add 0 points for each “yes” answer, for the answer “no” add 1 point.
Question 29: add 0 points for the answers “good” and “satisfactory”, and 1 point for the answers “bad” and “very bad”.
The sum of points received for all the answers is the indicator of SAH - it is entered in the formula.

Formulas for calculating of biological age (BA).

Men: BA = 27.0 + 0.22xSBP - 0.15xBH + 0.72xSAH - 0.15xSB
Women: BA = -1.46 + 0.42xPP + 0.25xBW + 0.70xSAH - 0.14xSB

Expected biological age (EBA)

Men: EBA = 0.629xCA + 18.6

Women: EBA = 0.581xCA + 17.3

CA is the calendar age.

The relative biological ageing index is biological age minus expected biological age (BA-EBA). A positive index value means that the biological age exceeds the expected biological age. Such people are biologically older than most of their peers. Negative values of the indicator correspond to the expected biological age over the biological age. Such people are biologically younger than most of their peers.

Online reference and more examples of calculation for men and women (in Russian) :

https://medbe.ru/materials/sportivnaya-reabilitatsiya/opredeleniya-biologicheskogo-vozrasta/

2. Subjective psychological age (PA) (self-assessment, according to K.A. Abulkhanova and T.N. Berezina).

Evaluate your psychological age on a 100-point scale: from 0 to 100. You can choose any number in this interval that corresponds to how you evaluate your psychological age.

0 points is the psychological age of a newborn baby who has neither life experience nor personality, whose psyche is just beginning to develop.

100 points is the psychological age of a person completing their life's journey, who has achieved everything or will never reach it, whose psyche is undergoing age-related degradation.

Choose any age in the range from 0 to 100, corresponding to your subjective sensation.

The resulting number is the psychological age of the person (in years).

If the indicator of psychological age is less than the calendar age, then such a person evaluates themself as psychologically younger in their course of life.

If the psychological age is more than the calendar age, then such a person evaluates themself as more mature.

If the psychological age coincides with the calendar age (with a difference of several years), then such a person evaluates their course of life as adequate to their age.

3. Index of relative psychological aging (PA-CA).

The relative psychological aging index is calculated by the formula:

Psychological age (PA) minus calendar age (CA). Negative values indicate psychological youth. Positive values - increased psychological aging.
